# 544 Southern US Burn Centers, Surge Capacity and 15 Months of the COVID19 Pandemic

**DOI:** 10.1093/jbcr/irac012.172

**Published:** 2022-03-23

**Authors:** Randy D Kearns, Carl A Flores, William L Hickerson, Jeffrey E Carter

**Affiliations:** University of New Orleans, New Orleans, Louisiana; University Medical Center New Orleans, New Orleans, Louisiana; Spectral MD, Avita Medical, AccessPro Med, Memphis, Tennessee; University Medical Center- New Orleans, New Orleans, Louisiana

## Abstract

**Introduction:**

Burn mass casualty incident (BMCI) planning efforts have been in practice and publication for 40+ years. Through these ongoing efforts, we know there are measurable limits to burn center capacity and capability through modeling and real-world events relying on conventional and contingency standards of care, even when the only focus is those patients with burn injuries. The southern region of the American Burn Association (ABA) includes 37 burn centers and continues to play a critical role in the BMCI preparedness process.

COVID-19 has emerged as the greatest pandemic in terms of morbidity and mortality since the 1918 influenza pandemic. While COVID-19 has no direct connection to burn injuries, the impact of COVID-19 on the American Healthcare System to include burn care was and remains significant.

**Methods:**

We conducted a retrospective analysis of (southern) regional data voluntarily submitted to the ABA from March 2020 to June 2021 and generally coincides with the first three waves of the pandemic. We focused on the self-reported data specific to the three critical components in managing a surge of patients: staffing, space, and supplies (to include pharmaceuticals and equipment).

**Results:**

Staff: These data were collected over a period that coincided with the first three waves seen in the region. Staffing shortages were noted during each of the surges but were most excessive when a regional surge paralleled surges in other parts of the country (November-December 2020).

**Space:**

Late November and early December 2020, space was in short supply with the surge of patients for more of the region than at any other time during the 28 weeks of reporting. While single facilities reported other episodes of limited space or supplemented with temporary structures, the peak was early December.

**Supplies:**

As the first surge began to subside, the supply shortages were abated. However, as additional surges occurred, the supply chain had not recovered. Supply shortages were reported in greater numbers than either space or staffing needs through the multiple waves of the pandemic.

**Conclusions:**

The surge of patients that had to be managed by the greater healthcare community placed a substantial strain on the burn centers to keep beds dedicated for patients with burn injuries. The pandemic directly led to a diminished available capacity for burn care in such a way that it could have compromised our ability to confront a surge of burn-injured patients. Future BMCI planning efforts must consider this aspect of the process. Crisis Standards of Care may come into play during such an event.

